# Robust Automated Amygdala Segmentation via Multi-Atlas Diffeomorphic Registration

**DOI:** 10.3389/fnins.2012.00166

**Published:** 2012-11-29

**Authors:** Jamie L. Hanson, Jung W. Suh, Brendon M. Nacewicz, Matthew J. Sutterer, Amelia A. Cayo, Diane E. Stodola, Cory A. Burghy, Hongzhi Wang, Brian B. Avants, Paul A. Yushkevich, Marilyn J. Essex, Seth D. Pollak, Richard J. Davidson

**Affiliations:** ^1^Department of Psychology, University of Wisconsin-MadisonMadison, WI, USA; ^2^Department of Radiology, University of PennsylvaniaPhiladelphia, PA, USA; ^3^Waisman Center, University of Wisconsin-MadisonMadison, WI, USA; ^4^University of IowaIowa City, IA, USA; ^5^Department of Psychiatry, University of Wisconsin-MadisonMadison, WI, USA

**Keywords:** amygdala, automated segmentation, structural MRI, amygdala volume, Freesurfer, medial temporal lobe, diffeomorphic warping, hand-tracing

## Abstract

Here, we describe a novel method for volumetric segmentation of the amygdala from MRI images collected from 35 human subjects. This approach is adapted from open-source techniques employed previously with the hippocampus (Suh et al., 2011; Wang et al., 2011a,b). Using multi-atlas segmentation and machine learning-based correction, we were able to produce automated amygdala segments with high Dice (Mean = 0.918 for the left amygdala; 0.916 for the right amygdala) and Jaccard coefficients (Mean = 0.850 for the left; 0.846 for the right) compared to rigorously hand-traced volumes. This automated routine also produced amygdala segments with high intra-class correlations (consistency = 0.830, absolute agreement = 0.819 for the left; consistency = 0.786, absolute agreement = 0.783 for the right) and bivariate (*r* = 0.831 for the left; *r* = 0.797 for the right) compared to hand-drawn amygdala. Our results are discussed in relation to other cutting-edge segmentation techniques, as well as commonly available approaches to amygdala segmentation (e.g., Freesurfer). We believe this new technique has broad application to research with large sample sizes for which amygdala quantification might be needed.

## Introduction

The amygdala has been shown to play a central role in emotion, along with various psychopathologies such as major depression, anxiety disorders, and autism (Hamilton et al., 2008; Stanfield et al., 2008; Hajek et al., 2009). Volumetric measurements have been one of the key metrics used to demonstrate a relationship between structure and function for this brain region (e.g., Campbell et al., 2004; Videbech and Ravnkilde, 2004; Nacewicz et al., 2006).

Volumetric quantification of the amygdala can be performed using manual and automated protocols. Manual hand-tracing is generally regarded as more accurate, but is often time-consuming and dependent on rater experience. With larger and larger sample sizes, automated segmentation has become more common (e.g., Bickart et al., 2011; Mattai et al., 2011; Butterworth et al., 2012). Such methods afford consistent quantification of medial temporal lobe structures, however the validity and accuracy of automated segmentation of the amygdala may be inconsistent. For the hippocampus, such methods can achieve high reproducibility and good accuracy, and are regarded as more efficient than hand-drawing (Tae et al., 2008; Morey et al., 2009). In regards to the amygdala, commonly available automated methods appear to yield unsatisfactory results with high-variability and low-validity (Babalola et al., 2009; Morey et al., 2009, 2010; Dewey et al., 2010). More rigorous approaches (e.g., Collins and Pruessner, 2010) still yield automated amygdala segments that could be improved and optimized with higher intra-class correlations and/or Dice coefficients.

Here, we detail a novel method for volumetric segmentation of the amygdala, adapted from open-source techniques employed previously with the hippocampus (Suh et al., 2011; Wang et al., 2011a,b). This approach consists of multi-atlas segmentation and machine learning-based correction. Multi-atlas segmentation involves registering training images from different subjects (structural MRI scans and corresponding manual segmentations) to MRI data where the segmentation is not known, or what we called test subjects (Aljabar et al., 2009). The resulting registration parameters are then used to propagate the known manual segmentation to the test (novel subject) data. With multiple training images, segmentation can be combined to improve accuracy. Information from these multiple images can be combined using “label fusion” strategies which involve voxel-by-voxel voting among the multiple training images. The success of segmentation is further increased when training images more similar to the test image receive a greater weight. Such approaches have been employed extensively (Klein et al., 2005; Heckemann et al., 2006; Aljabar et al., 2009), with recent research finding multi-atlas segmentation produced the best accuracy amongst four different automatic methods (Babalola et al., 2009; Leung et al., 2010).

Our approach also involves machine learning-based correction (AdaBoost), using a cross-validation strategy to train classifiers to recognize and correct the errors made by the multi-atlas segmentation relative to manual segments. Previous research on medial temporal lobe segmentation (e.g., hippocampus) has found that many of the common errors produced by multi-atlas fusion can be reduced using AdaBoost post-processing (Wang et al., 2011a; Wang and Yushkevich, 2012). Such corrective techniques were also recently shown to significantly improve multi-atlas fusion in an international challenge on brain segmentation with multi-atlas labeling (Landman and Warfield, 2012). In this paper, we first demonstrate how such a method yields high reproducibility and good accuracy for automated segmentation of the amygdala. We also show how these algorithms excel compared to currently available automated methods (e.g., Freesurfer – Fischl et al., 2002; Fischl et al., 2004).

## Methods

Our automated amygdala segmentation first involved the creation of a template and generation of multiple reference (or “training”) images. These training images were randomly chosen adults where rigorous amygdala hand-tracing was completed using a well-validated tracing protocol (i.e., Nacewicz et al., 2006). After the development of these files, we report the application of automated amygdala segmentation to novel (or “test”) subjects.

### Participants in training set

T1-weighted images were collected from 20 participants (Mean Age = 18.55 ± 0.16 years; eight female) using a GE 750 3T scanner equipped with high-speed gradients and an eight-channel receive-only phased-array head coil (GE Medical Systems, Waukesha, WI, USA). Three subjects met criteria for an anxiety disorder, all others were free of psychopathology. All subjects were free of neurological disease/neurodegenerative disorders. These anatomical images were high-resolution 3-D, inversion recovery prepped fast spin-echo images with the following parameters: TE = 1.8 ms, TR = 8.9 ms, field of view (FOV) = 240 mm × 240 mm, flip angle = 10°, matrix = 240 × 240, 124 axial slices, slice thickness = 1.0 mm.

### Amygdala hand-tracing procedures

As detailed in Nacewicz et al. (2006, 2012), T1-weighted images from these 20 participants were skull-stripped (Cox, 1996), and corrected for intensity bias using tissue segmentation with spatial priors in FSL’s FAST routine^1^. The resultant image was automatically scaled (contrast adjusted) so that the peak of the white matter histogram (mode of white matter intensity) was at 80% of the 16-bit range using in-house software (written in Python) and AFNI’s 3dcalc tool. Image intensities were then squared for optimal peak separation and contrast adjustment was repeated. The resultant images were then segmented using FAST with spatial priors. This procedure produces images with superb separation of all tissue types and optimizes contrast for viewing variations in the gray matter distribution.

Amygdala ROIs were manually traced by baccalaureate-level scientists (MJS and AAC) with extensive training in temporal lobe neuroanatomy, according to established protocol (Nacewicz et al., 2006). Hand-tracing took approximately 2 hours per amygdala per subject to complete, with one subject taking 4–5 hours for bilateral amygdala quantification. Briefly, the optic tract, optic radiations, hippocampus, and inferior horn of the lateral ventricle defined posterior borders; temporal lobe white matter, cerebrospinal fluid (CSF), anterior commissure, and entorhinal cortex defined anterior boundaries. Following initial tracing in the axial plane, the sagittal view was used to confirm accurate separation of the amygdala from hippocampus, entorhinal cortex, optic radiations, and caudate/putamen; coronal view was used for refinement of the dorsolateral and inferomedial boundaries.

Region of interest drawing of the amygdala was completed using in-house software (Spamalize)^2^ that allows for simultaneous visualization and ROI definition in the three cardinal planes. All tracing was carried out by raters blind to group, using a technique that was highly reliable (an inter-rater intra-class correlation = 0.95 for volume and a high-spatial reliability: mean Jaccard coefficient = 0.84).

### Novel (or test) sample used for segmentation validation

T1-weighted images were collected from 35 participants (Mean Age = 18.63 ± 0.28 years; 19 female) again using a GE 750 3T scanner equipped with same parameters specified above. Three subjects met criteria for anxiety disorders, five subjects met criteria for substance dependence (three alcohol, two cannabis), and all others were free of psychopathology. All subjects were free of neurological disease/neurodegenerative disorders. These subjects also had amygdala tracing completed on their T1-weighted images but were not used in template creation. This tracing was completed to serve as a comparison to our volumes generated by our automated approach.

### AHEAD segmentation

**Figure 1:**
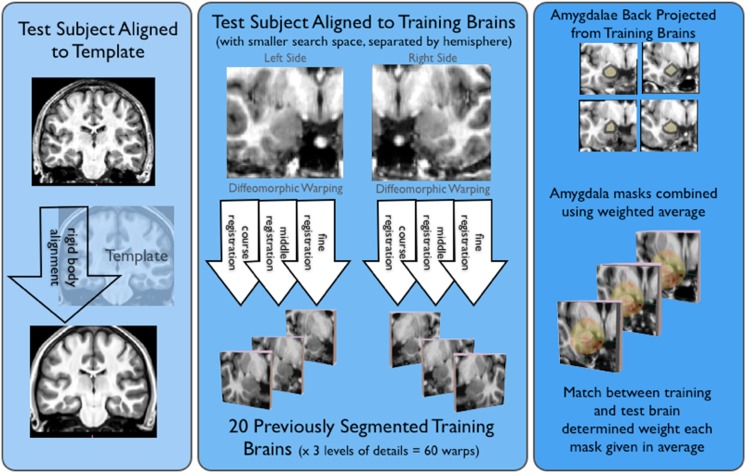
**Shows the basic AHEAD processing pipeline**. For the first step of the pipeline shown on the left side of this image, subjects were aligned to our template via a rigid body transform. Next, shown in the middle of the figure test subjects were aligned to our training brains. This yielded 60 warps for both the left and right amygdala (20 training brains × three levels of detail). As shown on the right side of the figure, amygdala masks were projected from the training brains and combined in a weighted average. The match between training and test determined the weighting for each amygdala mask.

Automatic Hippocampal Estimation using Atlas-based Delineation (AHEAD)^3^ algorithm detailed in Suh et al. (2011) was adapted to automatically segment amygdalae. As shown in Figure 1, this approach involved alignment to a T1-weighted template (to define a smaller search space for our ROIs), diffeomorphic warping using our “training” brains, and then weighting and averaging the resulting amygdala masks, for all novel (or test) subjects.

**Figure 2:**
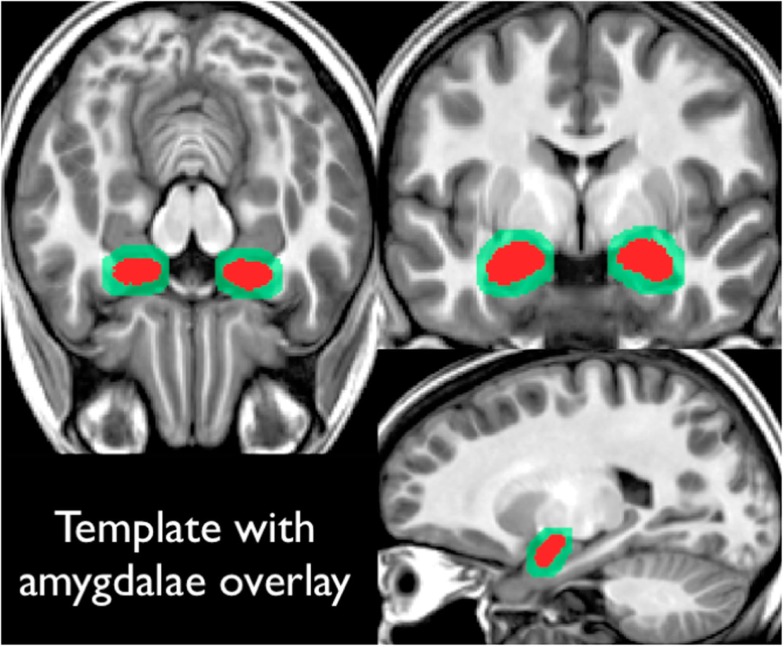
**Shows the high-resolution template constructed from our 20 training subjects**. All subjects had T1-weighted structural images along amygdala region of interest drawings. Region of interest drawings were transformed into this template space and summed (shown in red on this figure). This summed amygdala mask was then smoothed with a 1 mm^3^ Gaussian filter (as shown in light green in this figure).

Our scanner specific T1-weighted template was created through an iterative averaging and diffeomorphic warping from the 20 (training) subjects referenced previously (final template shown in Figure 2). These subjects had high-resolution T1-weight scans, along with amygdala masks drawn by hand. Each subject’s amygdala masks were transformed into this template space using the warping parameters calculated from the T1-registration. This yielded a summed amygdala image that was smoothed using a 1-mm^3^ Gaussian filter.

**Figure 3:**
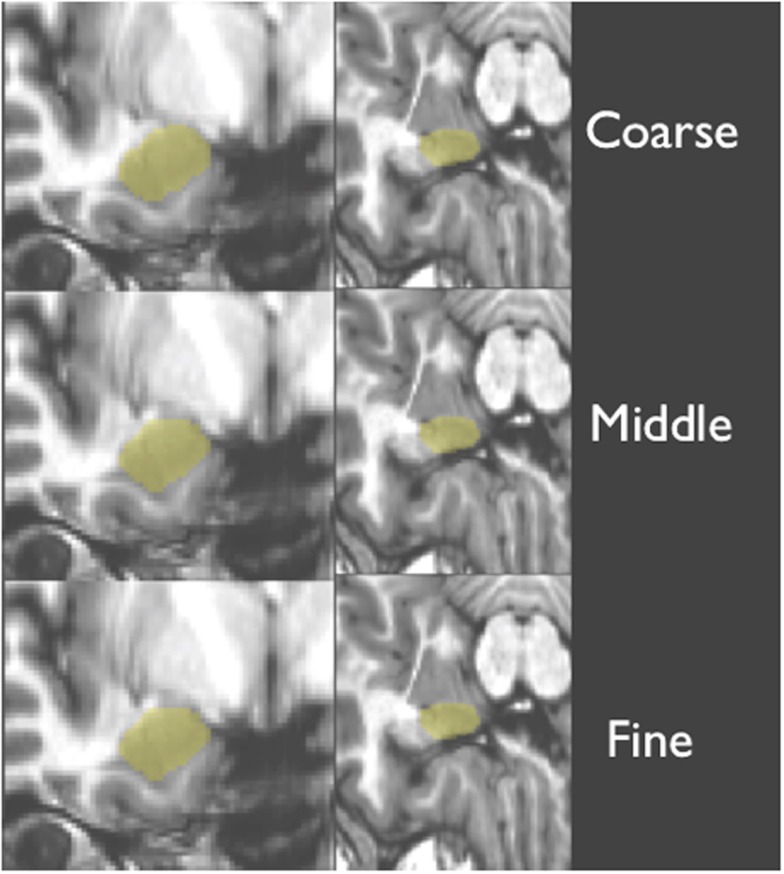
**Shows a structural image from a novel subject, displaying the three levels of detail involved with warping and amygdala mask back projection (overlaid in yellow)**.

T1-weighted structural scans for each novel (or test) subject were first bias corrected to minimize field inhomogeneity. Next, T1-weighted images of novel (test) subjects were deformably registered to a T1-weighted population template via Symmetric Normalization (SyN: Avants and Gee, 2004). SyN was found to be one of the top two performers in a recent evaluation study of 14 open-source deformable registration algorithms by Klein et al. (2009). This step factored out much of the anatomical differences between subjects and put all scans into a common space for subsequent steps of our processing pipeline. Next, a ROI surrounding of the amygdala in each hemisphere was defined automatically using an average ROI, producing a smaller search space for our later steps. Then, the T1-weighted structural scan from each novel subject was warped with three increasing levels of detail (coarse, middle, and fine, as shown in Figure 3) to each one of our training brains (the 20 subjects specified above). This registration employed a maximum of 80 iterations at 4x subsampling (coarse), 80 iterations at 2x subsampling (middle), and 30 iterations at full resolution (fine).

After this, consensus segmentation using similarity-weighted voting was completed. This approach allows for the contribution from each training segment to be weighted locally by the image match between the T1-weighted image of the test subject and the T1-weighted image of the training subject. The scheme is local because voting occurs independently at each voxel. Additional details regarding this step are discussed in Wang et al. (2012).

The initial segmentations produced using this pipeline were further refined using a segmentation error correction strategy (Wang et al., 2011a) that employs AdaBoost (Freund and Schapire, 1995). Initial segmentations produced by our processing pipeline were compared to the ground truth manual segmentations, and mislabeled voxels were identified. This correction step involves two basic components-bias detection and bias correction. Bias detection involves finding systematic biases in the initial segmentation relative to the ground truth, whereas bias correction simply involves adjusting mislabeled voxels based on appearance-, contextual-, and spatial-features. This procedure is explored in greater depth in Wang et al. (2011a), as well as Yushkevich et al. (2010). Using these features, candidate voxels suspected to be mislabeled in the initial segmentation were ouput and relabeled. In total, generation of amygdala segments took approximately 4 hours per subject, using a AMD Quad Core 2384 computer with 16 GB memory and a CPU of 2.7 GHz.

### Automated segmentation routine with freesurfer

For comparison purposes, segmentation of the amygdala was also performed for our 35 novel subjects in the Freesurfer image analysis suite, which is documented and freely available for download online^4^. Briefly, this processing includes motion correction, removal of non-brain tissue using a hybrid watershed/surface deformation procedure (Ségonne et al., 2004), automated Talairach transformation, segmentation of the subcortical white matter, and deep gray matter volumetric structures (including the amygdala; Fischl et al., 2002, 2004).

## Results

**Figure 4:**
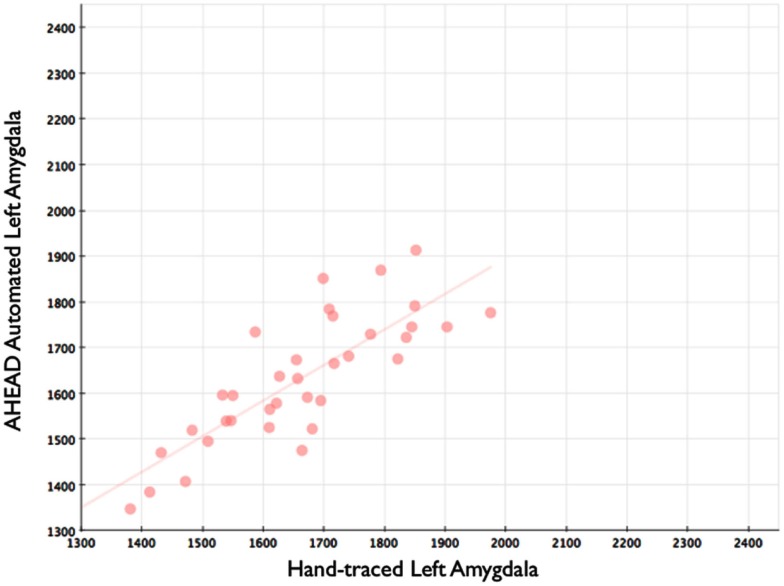
**Shows a scatterplot with automated segments of the left amygdala generated by our approach on the horizontal axis, and hand-drawn left amygdalae on the vertical axis**.

**Figure 5:**
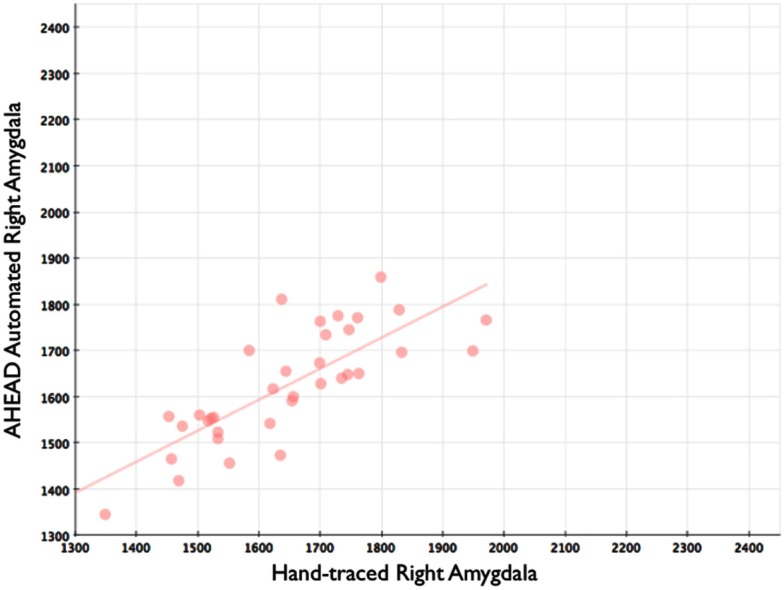
**Shows a scatterplot with automated segments of the right amygdala generated by our approach on the horizontal axis, and hand-drawn right amygdalae on the vertical axis**.

**Figure 6:**
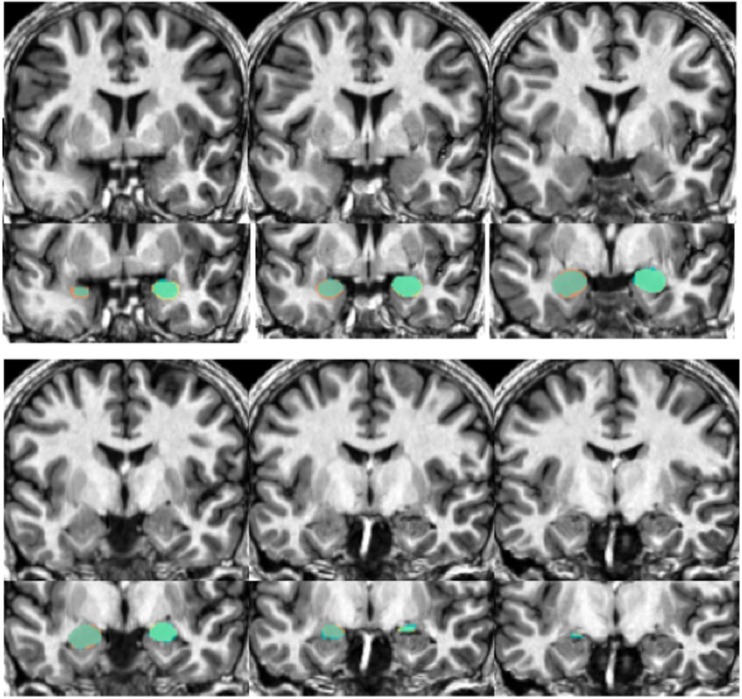
**Shows an example structural image from a novel subject (top portion of each section) with amygdalae overlaid (bottom portion)**. Slices move anterior to posterior, with the top left showing more anterior portions of the amygdala while the bottom right show more posterior sections. The overlap between automated segments generated from our approach and hand-drawn volumes appear in aqua. Portions of hand-drawn amygdala volumes not capture by automated segmentation appear in orange (for the right) and yellow (for the left amygdala).

Our automated amygdala segmentation of novel subjects yielded high-agreement with our hand-drawn amygdala volumes conducted on the same participants. The bivariate correlations between hand-drawn amygdalae and our automatically generated volumes was high (Left: *r* = 0.831, *p* < 0.001; Right: *r* = 0.797, *p* < 0.001). Scatterplots of these relationships are shown in Figures 4 and 5. Intra-class correlations between hand-drawn amygdalae and our automatically generated volumes were also high [Left ICC (for consistency) = 0.830; Left ICC (for absolute agreement) = 0.819; Right ICC (for consistency) = 0.786; Right ICC (for absolute agreement) = 0.783]. Amygdala segments generated from this automated routine also demonstrated high-spatial reliability. Dice coefficients (comparing our hand-traces and our automated volumes) for the left amygdala were 0.918 ± 0.035, with mean Jaccard coefficients of 0.850 ± 0.055. For the right amygdala, similar results were found with 0.916 ± 0.032, with mean Jaccard coefficients of 0.846 ± 0.053. Shown in Figure 6 is an example T1-weighted image with amygdalae drawn by hand and segments generated from our novel approach.

**Figure 7:**
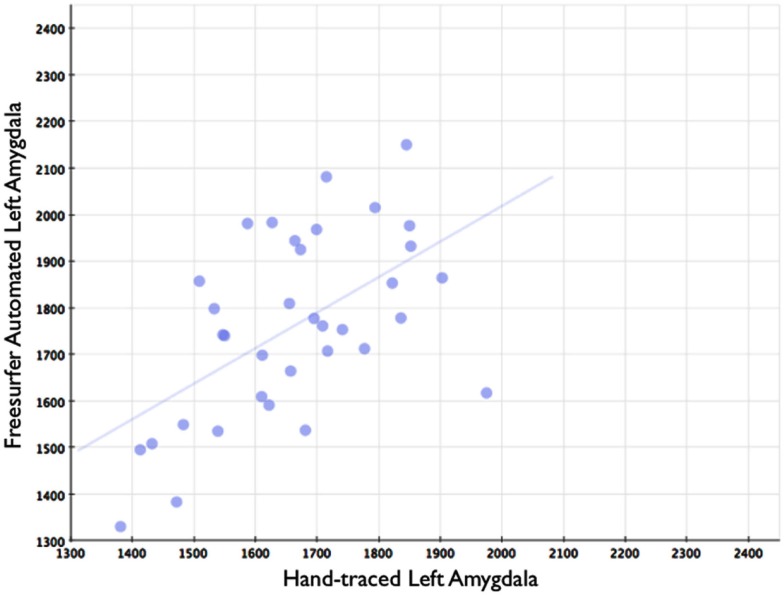
**Shows a scatterplot with segments of the left amygdala generated by Freesurfer on the horizontal axis, and hand-drawn left amygdalae on the vertical axis**.

**Figure 8:**
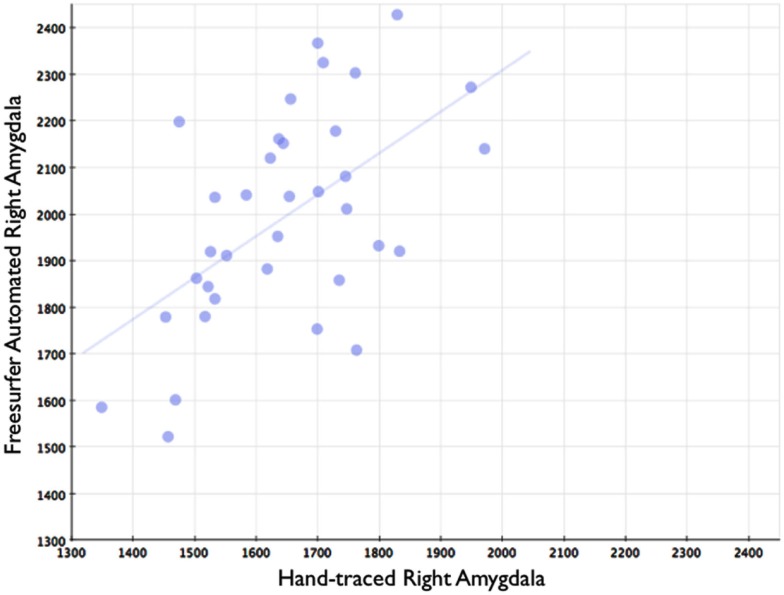
**Shows a scatterplot with segments of the right amygdala generated by Freesurfer on the horizontal axis, and hand-drawn right amygdalae on the vertical axis**.

**Figure 9:**
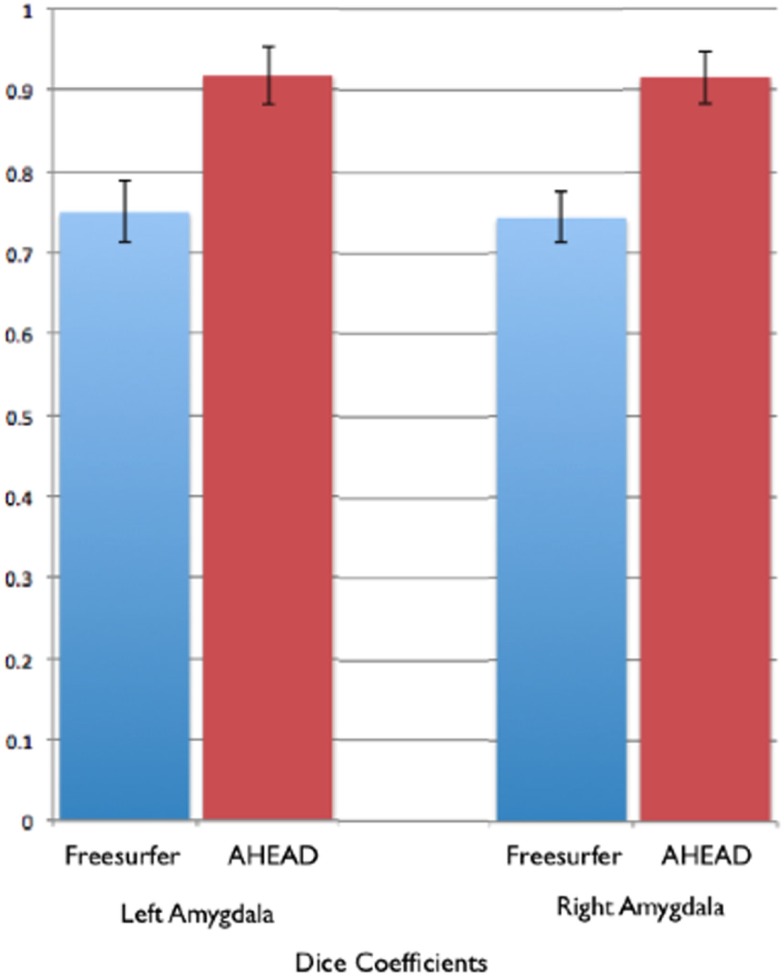
**Shows bar graphs of the spatial statistics (Dice coefficients) for automated amygdala using the AHEAD processing pipeline (in red) and for Freesurfer (in blue)**. These spatial statistics were generated by examining the amount of spatial overlap between automated amygdala segments generated by each, compared to our rigorous hand-tracing amygdala routine.

In comparison, automated segments of the amygdala generated by Freesurfer had lower bivariate correlations (Left: *r* = 0.563, *p* < 0.001; Right: *r* = 0.560, *p* < 0.001). Scatterplots for these volumes are shown in Figures 7 and 8. Intra-class correlations were also lower between Freesurfer and our hand-drawn amygdalae [Left ICC (for consistency) = 0.537; Left ICC (for absolute agreement) = 0.468; Right ICC (for consistency) = 0.504; Right ICC (for absolute agreement) = 0.189]. Freesurfer produced automated amygdala segments with lower spatial reliability. Dice coefficients (comparing our hand-traces and Freesurfer automated volumes) for the left amygdala were 0.749 ± 0.03, with mean Jaccard coefficients of 0.601 ± 0.046. For the right amygdala, similar results were found with 0.743 ± 0.03, with mean Jaccard coefficients of 0.592 ± 0.04. These statistics for Freesurfer are similar to previous reports (e.g., Morey et al., 2009). A comparison of Freesurfer and our novel algorithm is shown Figure 9.

## Discussion

We demonstrate here that reliable and highly valid segmentation of the amygdala can be achieved via automated algorithms that rely on multi-atlas diffeomorphic registration followed by label fusion. Such procedures yield segments that have high bivariate correlations, intra-class correlation, and spatial overlap with hand-drawn amygdala segments. Such methods have broad applications in addressing a wide range of questions in basic affective neuroscience research, neurobiological studies of major depression, and studies on the neural bases of autism. We also demonstrate here that these novel methods outperform conventional publicly available methods of automated segmentation of amygdala (i.e., Freesurfer), providing a more valid approach to the study of the medial temporal lobe.

Previous research (Morey et al., 2009) has found other publicly available methods of amygdala segmentation (e.g., FMRIB Integrated Registration and Segmentation Tool) correlate poorly with manual tracing; this motivated our use of Freesurfer as a comparison. Future research should attempt to compare our novel method with cutting-edge techniques such as automatic non-linear image matching and anatomical labeling with label fusion (e.g., Collins and Pruessner, 2010). Many of these methods are however not currently publicly available (Personal communication, Louis Collins, October 3, 2012).

Of important note, subtle differences exist in the amygdala quantification routines employed in our automated processing and with other tools (e.g., Freesurfer). Different boundaries and landmarks may explain a portion of the differences seen across the tools. Future research is needed to compare across routines, to aid in determining the tracing routine most appropriate for medial temporal lobe quantification. Future research could also expand on this work and aim to automatically segment subregions of the amygdala based on existing protocols (e.g., Saygin et al., 2011; Solano-Castiella et al., 2011; Entis et al., 2012).

This approach also has a number of important limitations that must be considered before universal implementation. We had access to highly reliable and rigorously quantified amygdala drawing acquired on a GE 750 scanner. Future applications of such a technique to novel subjects should employ training data that most closely matches new scans to be segmented. A fair number of training subjects (20) were employed in this study that other research groups may not have access to. All training subjects were adults; additional training subjects with greater variability in age and other demographic factors may led to a wider application of this novel segmentation approach.

To our knowledge, this is one of the first studies to obtain highly valid automated segmentation of the amygdala. Few previous research reports have compared automatic methods to such rigorously defined hand-drawn routines (e.g., hand-tracing with spatial reliability <0.8 and inter-rater intra-class correlation <0.9). Based on these findings, we believe this new technique has broad application to research that has begun to employ increasingly large sample sizes.

## Conflict of Interest Statement

The authors declare that the research was conducted in the absence of any commercial or financial relationships that could be construed as a potential conflict of interest.

## Footnotes

^1^http://www.fmrib.ox.ac.uk/fsl

^2^http://psyphz.psych.wisc.edu/~oakes/spam/spam_frames.htm

^3^http://www.nitrc.org/projects/ahead

^4^http://surfer.nmr.mgh.harvard.edu/
